# All-inorganic perovskite nanocrystals: next-generation scintillation materials for high-resolution X-ray imaging

**DOI:** 10.1039/d1na00815c

**Published:** 2021-12-30

**Authors:** Lu Lu, Mingzi Sun, Tong Wu, Qiuyang Lu, Baian Chen, Bolong Huang

**Affiliations:** Department of Applied Biology and Chemical Technology, The Hong Kong Polytechnic University Hung Hom Kowloon Hong Kong SAR China bhuang@polyu.edu.hk

## Abstract

With super strong penetrability, high-energy X-rays can be applied to probe the inner structure of target objects under nondestructive situations. Scintillation materials can down-convert X-rays into visible light, enabling the reception of photon signals and photoelectric conversion by common sensing arrays such as photomultiplier tubes and amorphous-Si photodiode matrixes. All-inorganic perovskite nanocrystals are emerging photovoltaic and scintillation materials, with tremendous light-conversion efficiency and tunable luminous properties, exhibiting great potential for high-quality X-ray imaging. Recent advancements in nanotechnology further accelerate the performance improvement of scintillation materials. In this review, we will provide a comprehensive overview of novel all-inorganic perovskite nano-scintillators in terms of potential applications in low-dose X-ray medical radiography. Compared with conventional scintillators, the merits/drawbacks, challenges, and scintillation performance control will be the focus of this article.

## Abstract 2

This review summarizes the recent research progress in all-inorganic perovskite nano-scintillators in terms of their potential applications in X-ray imaging and provides references on scintillation performance control for future material design.

## Graphical abstract

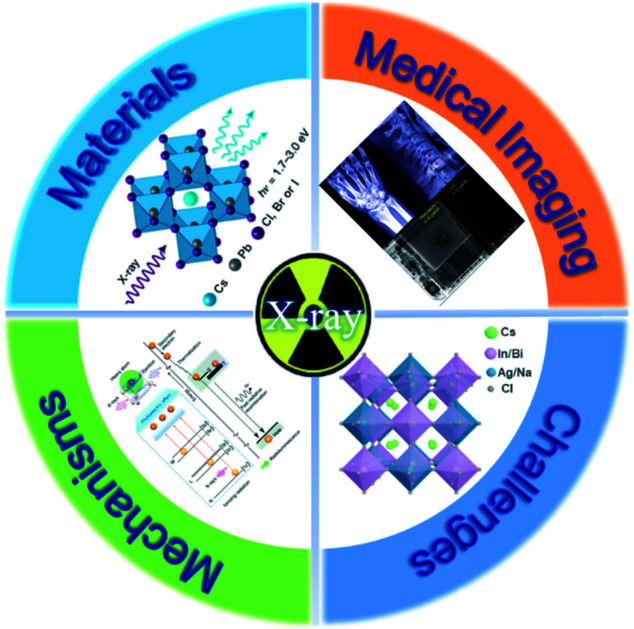

## 1. Introduction

X-ray is a kind of high energy electromagnetic radiation with ultra-strong penetrability, which can probe the inner situations under nondestructive testing.^1^ It was first discovered by Wilhelm Röntgen in the year 1895,^2^ and has been widely used in multiple fields such as medical radiography, photodynamic therapy (PDT), nuclear prevention and security inspection in recent decades.^3^ The fundamental working principle of X-ray sensing is to monitor and reveal the attenuation of incident X-ray radiation after penetrating the specific targets. For instance, the intravital organ penetrating makes high-resolution radiography, which has been widely used in our daily life such as clinical diagnosis. The non-living objects penetrating enables industrial and security applications.^4^ Currently, the modern X-ray detection techniques are generally classified into two categories: direct conversion from X-ray quanta to electrical signal *via* a semiconductor or to chemical signal *via* a film; and indirect conversion to low-energy photons (visible light) by scintillating materials.^1,5,6^

Up to now, for general X-ray imaging with integrating detectors, there are hardly any direct converting materials that can commercially replace nano-scintillators except for selenium (Se) detector for mammography.^6^ Even the most competitive direct conversion materials with high resolution,^7^ CdTe and CdZnTe (CZT), still suffer from low response rate and temporal effects such as long afterglow caused by electron trapping.^1,6^ Also, it is still difficult to manufacture a large-size wafer with a low noise current but high mobility-lifetime-product (*μτ*), making it tough to achieve the acceptable price to performance ratio for commercial applications.^8^ By contrast, scintillators can convert X-ray into visible light (down conversion), enabling the commercial applications of low-cost sensing arrays like photomultiplier tube (PMT), avalanche photodiode (APD),^9^ amorphous-Si photodiode matrix, and charge-coupled devices.^10^ Thus, emerging indirect conversion scintillating materials have attracted more research interests for X-ray sensing.

Conventional scintillators like NaI:Tl^+^,^11,12^ CsI:Tl^+^, CdWO_4_ (CWO),^13,14^ Bi_4_Ge_3_O_12_ (BGO),^11,12^ Lu_2_SiO_5_ (LSO), and Lu_2(1−*X*)_Y_2*X*_SiO_5_ (LYSO) have been commercially applied in medical imaging devices due to their high light output and strong stopping power. However, these materials still require further optimization to simplify the manufacturing processes, avoid the afterglow and achieve tunable scintillation by finding new electron-transition energy levels.^15–17^ Especially for diagnostic radiology, realizing higher spatial resolution under lower dose radiation exposure is considered as the foremost appeal.^18,19^ Owing to the attractive merits including easier fabrication, faster response, and higher spatial resolution, lead-halide perovskite nanocrystals based X-ray sensors are emerging to exhibit great application prospects in future radiography.^15,20^ In terms of electronic properties, these halide perovskites usually have lower trap density,^21^ higher charge-carrier mobility^22^ and longer minority-carrier diffusion length,^23^ peculiarly in nanocrystal morphology.^4,24,25^

With a fast response in a nanosecond, CsPbCl_3_ was observed to have strong luminescence by free excitons when exposed to radiation, making it a promising scintillation material for X-ray detection in the late 20th century.^26^ Whereas, the strong self-absorption, low photoluminescence quantum yield (PLQY), the toxicity of Pb, and poor stability of Pb-halide perovskites greatly limit their large-scale industrialization for X-ray medical imaging.^27^ After decades of research, a series of Pb-free scintillators such as double-perovskite,^28,29^ Cu,^30,31^ and Bi^32^-based all-inorganic halide perovskites have been discovered that showed satisfying scintillation behaviors. For example, Rb_2_CuBr_3_ (ref. 30) and Cs_2_NaTbCl_6_ (ref. 33) are popular Pb-free scintillators with high PLQY, however, the unfavored afterglow emission often causes reduced imaging contrast, and the impact is even worse in computed tomography (CT). The emission band of Rb_2_CuBr_3_ is peaked in the blue light range, not in the maximum sensitivity window of silicon photodetectors (peak photo-response in 700–900 nm). Thus, for realizing high contrast X-ray imaging, the exploration of Pb-free halide perovskite nano-scintillators with fast decay time and matched emission wavelength has been a recent research hotspot.

In this work, we have supplied a comprehensive review of recent achievements in the new generation of all-inorganic perovskite nano-scintillators for high-quality X-ray imaging. Firstly, the general working principle of X-ray detectors, scintillation mechanisms, and application criteria of scintillators for high resolution but low dose exposure X-ray imaging will be stated in detail. The mechanism of scintillation performance control based on native defects is one of the focuses. Then, the main merits/drawbacks, growth methods, performance, and applications of the emerging halide perovskite nano-scintillators are clarified. Recent breakthroughs and modification strategies on scintillation performance are demonstrated systematically. Comparisons of scintillation performance have been made between traditional and novel scintillators. Finally, we propose the remaining challenges of current all-inorganic perovskite nanocrystal scintillators for low-dose X-ray imaging to reach commercial applications. This review will provide insightful ideas for future scintillation material design and optimizations.

## 2. Scintillation mechanism

### 2.1 Working principle of X-ray detectors

**Fig. 1:**
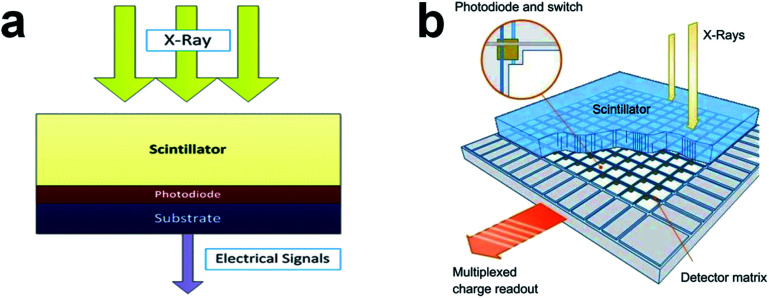
(a) Schematic depiction of the three main functional layers in a common X-ray CT detector. Reproduced with permission.^34^ Copyright 2013 Springer Nature Limited. (b) Schematic description of an X-ray flat detector constituted by columnar CsI:Tl^+^ scintillator and a detecting matrix of amorphous-silicon photodiodes. Reproduced with permission.^18^ Copyright 2013 Elsevier.

Two conversion processes are mainly involved in the detection of X-ray: down-conversion of X-ray and photovoltaic conversion. As illustrated in Fig. 1a,^34^ most commercial X-ray detectors share a universal design with three main functional layers: scintillator (X-ray absorption and conversion), photodiode arrays (photovoltaic conversion), and a substrate (for mechanical and electrical installation and keeping the structure flat). Firstly, the incident X-ray will be absorbed and converted into visible light in the scintillation layer, and the emission photons are further transformed into electric current in the photodiode matrix. The inversely biased photodiode will be pre-charged at a certain voltage and afterward discharged due to the photoinduced electron–hole pairs generated by the proton collision. The generated electrical signal will be transmitted to analog electronic devices for signal amplification in the substrate layer. At last, the magnified signal will be digitized and subsequently delivered from the detecting platform to acquisition circuitry for the formation of images.

The schematic framework of the frequently used amorphous-Si flat detector is indicated in Fig. 1b.^18^ Generally, the scintillating material is CsI:Tl^+^ due to the superior scintillation properties including matching emission spectrum with amorphous-silicon photodiodes, high absorption coefficient, and high light output. Through the vapor deposition, the CsI:Tl^+^ scintillating crystal is formed in a needle-like structure in 5–10 μm diameter, with a length of ≥600 μm. This morphology can promote the collecting of emitted photons in the Si-photodiode matrix conjoined below. For modern multi-slice spiral CT scanners, an ultra-fast decay is the most important scintillation parameter. High-*Z*_eff_ scintillation crystals with a short afterglow lifetime like Gd_2_O_2_S (GOS) and Ce^3+^ doped Lu_3_Al_5_O_12_ (LuAG:Ce^3+^) are preferable for X-ray CT imaging.^18,35^ Different from common flat detectors, a more discrete structure for scintillation layer construction is allowed in CT imaging with a relatively large pixel size so the scintillators are separated in angular and axial orientation. As a result, it enables the optical separation for individual pixels and at the same time spatially fits the Si-photodiode arrays.

### 2.2 Scintillation mechanism of common scintillating materials

**Fig. 2:**
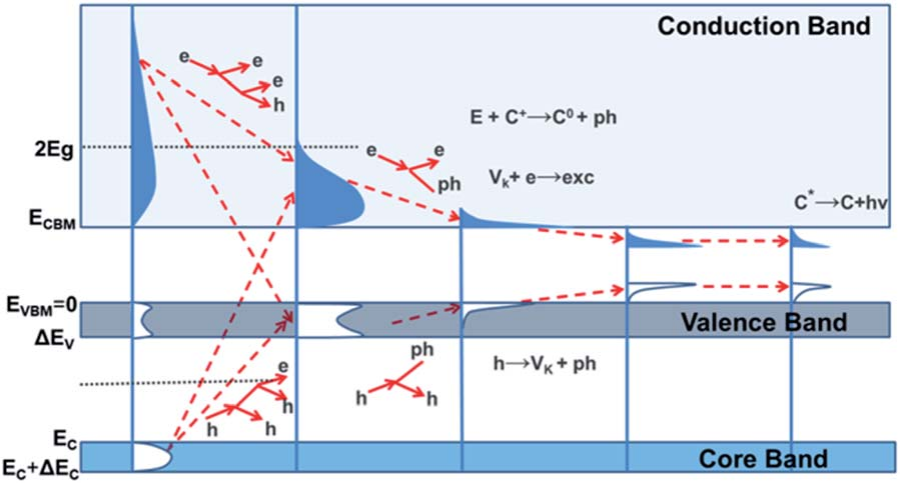
Schematic mechanism explained by the relaxation of electron excitations in inorganic scintillation crystals. *E*_C_ refers to the top energy of core band, and Δ*E*_C_ represents the corresponding bandwidth; *E*_VBM_ refers to the top energy of VB (*E*_VBM_ = 0), and Δ*E*_V_ represents the bandwidth of VB; *E*_CBM_ refers to the bottom of CB, and *E*_g_ represents the bandgap. *V*_k_ means self-trapped holes, and C^+^ represents ionic centers. Reproduced with permission.^36^ Copyright 2017 Springer Nature Limited.

Having a comprehensive understanding of the scintillating mechanism will benefit the optimization of existing scintillators as well as the design of next-generation scintillation materials. New experimental findings and emerging theoretical simulations will continuously benefit the advances of scintillating theory. Based on current findings and theoretical models, five main stages are involved in the whole scintillation process from initial X-ray excitation to final visible-light emission, and the schematic scintillation mechanism has been presented in Fig. 2.^36^

In the first stage, the initial excitations are produced by the interaction between ionizing particles and scintillators. The X-ray photons possess high energy, leading to the generation of deep holes (h^+^) and hot electrons (e^−^) in the inner core band and conduction band (CB), respectively. Followed by inelastic e–e scattering and auger process, h^+^ in VB/core band and e^−^ in CB will be created rapidly by massive secondary electron excitation. The multiplicative stimulation for charge-carrier creation will be terminated at the end of this step. Along with the creation of phonons, the thermalization of electron excitation is the main concern in the next stage, resulting in the generation of low kinetic-energy e^−^ in CB minimum (CBM) and h^+^ in VB maximum (VBM). Since the localization of electron excitation is usually accompanied by the replacement of atoms and photo-induced desorption, in the third stage, its interaction with native defects and external dopants/impurities in host lattice is the main feature. In the last two stages, charge carrier migration and the following radiative/nonradiative electron–hole recombination process is mainly involved. After final excitation *via* sequential charge-carrier capture or energy-transfer process, the last scintillation stage ends in the visible photon emission at the luminescent center. The interaction between electron excitations may cause reduction in their quantity, and it is considered as a decisive factor on material density-effect, nonexponential decay as well as nonproportional energy response.^36^

### 2.3 Scintillation performance control based on native defects

The electronic properties of defect states have strong impacts on material radiation stability and the production of persistent luminescence (PL). The formation of defects in host lattice is identified as the dominant cause for non-radiative energy losses in the scintillation process, including external dopants/impurities as well as intrinsic imperfections such as simple point defects (vacancies and interstitials), interfacial/surface states, and grain boundaries. Vacancy defects are very likely to be formed in fabrication processes particularly in heat treatments like annealing. Besides intrinsic point defects, the interaction between incident X-ray radiation and scintillation material also leads to the generation of lattice imperfection. Under X-ray excitation, with the vacancy formation in anionic sublattice, it is very common for inorganic oxide scintillators to have defects particularly when the dopant/impurity ions have different charge states from those in initial lattice sites. If the electronic energy states of defects appear below CBM and with a certain trap depth, the photo-induced electrons will be trapped and have delayed recombination with the relaxed holes at the luminescent center.^37^ The prolonged scintillation decay time will cause a severe negative influence on imaging contrast in high-speed CT scanning.

Having severe impacts on thermal conductivity and ionic conduction,^38–40^ the anion Frenkel (a-Fr) defects serve as the dominant imperfection type in fluorides and oxides lattice, formed by an anion moving from its original site to a neighboring interstitial site, resulting in an uncharged vacancy-interstitial defect pair in the lattice.^38,39,41,42^ The migrating charge carriers can be trapped and stored in these defect states, mainly in those of vacancies. The electrons/holes in traps will be released by ambient thermal disturbance, followed by phonon-assisted migration.^43^ It is energetically preferable for these electrons/holes to be transferred from the de-localized CB to localized states within the bandgap *via* phonon coupling, which leads to decreased transmitting rate of polarons. Thus, by restraining the formation of defects, the mobility of charge carriers can be accelerated, and the recombination delay, as well as the non-radiative energy losses will be shortened. The advancements in crystal growth technology and optimization in material morphology are expected to suppress the emergence of imperfections in host lattice, so the scintillating transport state can be significantly tuned by material manufacturing process control.^44^

It has been reported that the addition of co-dopants can suppress afterglow emission,^45–47^ but with drawbacks in lowered light yield.^48^ For instance, Ce^3+^ co-dopant can efficiently restrain the afterglow emission of GOS:Pr^3+^ and GOS:Tb^3+^, but it suffers a remarkable reduction in light output at relatively low temperatures.^49^ It should be noted that the addition of co-dopant ions will make an intentional compensation for intrinsic defects, but the total concentration of defects cannot be decreased. The lattice imperfections that do not have impacts on scintillating properties are expected to displace those detrimental ones under specific conditions. Thus, the potential influences that the dopants may make on defect formation should be fully understood for further scintillation performance modulation.^46^

Modern computational simulation technology can well reveal the structural and electronic properties of defects, so it is very promising for the design and performance optimization of next-generation scintillators. For example, the NaYF_4_ is a potential scintillation material, but its long afterglow will limit its application in high-resolution CT imaging.^3^ In our previous work, to reveal the PL mechanism of NaYF_4_, the electronic defect states in the bandgap are fully investigated by DFT calculation under the CASTEP code.^50^ The PL can be suppressed by facilitating the non-radiative recombination by applying specific doping schemes or defect states modulation based bandgap engineering.^3,47,51,52^ Based on the electronic properties including the distribution of defect states in host bandgap, the computational simulation can provide researchers with comprehensive theoretical support on finding potential co-dopants for PL elimination.

## 3. Requirements for X-ray scintillation imaging

Based on their unique down-conversion capacity (converting high-energy X-ray/γ-ray into visible light), scintillators have been widely used in nuclear security,^16^ medical imageology,^53–55^ and PDT.^56,57^ Since the intrinsic factors of scintillators have a direct impact on detection efficiency, the performance parameter requirements for these scintillating materials vary from different application fields. For the applications in homeland security, diffusely deployed sensing systems for γ-ray spectroscopy are the fundamental requirement. Consequently, energy resolution becomes the main concern. For X-ray CT imaging, current challenges focus on achieving higher conversion efficiency and spatial resolution, faster response, and avoiding afterglow for scintillator development. To this end, the key performance parameters of scintillators and the corresponding effects on detection efficiency, especially in terms of CT imaging, are discussed in the following sections.

### 3.1 Main parameters of common scintillators

#### (1) Conversion efficiency

The quantity of emission visible-light photon (*N*_ph_) generated during scintillating conversion process by per energy (*E*) of incident X-ray photon is expressed by the following formula:^58^

1 *N*_ph_ = {*E*/(*βE*_g_)}*SQ*

*β* is the phenomenological parameter, generally in the range of 2–3; *E*_g_ represents band-gap value; *S* refers to transfer quantum efficiency; and *Q* is the quantum efficiency of emission center. The corresponding efficiency *η* has been expressed by the formula as follows, where *E*_vis_ stands for the photon energy of emission visible light.^58^

2 *η* = *E*_vis_*N*_ph_/*E*

#### (2) Light yield (LY)

LY (in photons per MeV) is a key parameter for the description of scintillating efficiency, can be expressed by the following equation:^36^

3 LY = (10^6^/*βE*_g_)*SQ*

in which *E*_g_ represents the bandgap value (in eV), *β* = *E*_e–h_/*E*_g_, *E*_e–h_ represents the energy consumption for producing an electron–hole pair; *S* and *Q* refer to the quantum efficiency of transport and luminescence stage respectively.

#### (3) Decay time

The decay time is the duration time that the scintillators emit ultraviolet (UV)/visible light after the absorption of the incident X-ray energy. Short decay/fast response is essential for fast-scan imaging. In general, the quenching process can reduce decay time but cause a decline in LY.

#### (4) Stopping power

With fixed thickness, the attenuation coefficient relies on material density (*ρ*) and atomic number (*Z*_eff_). Thus, the stopping power is in direct proportion to *ρZ*_eff_.^58^

#### (5) Matching emission spectrum with the photodetector

To reduce post-scintillating loss, the radioluminescence spectrum should be well overlapped by the peak response window of the photodetector. For instance, scintillation emission in the visible/near-infrared (NIR) region to match the optimal detection sensitivity of silicon photodetector.

#### (6) Chemical stability

The chemical stability is largely affected by hygroscopicity, which is a key parameter that evaluates the material lifespan.

#### (7) Radiation hardness

This indicates the capacity that the scintillating performance remains undecayed under high-energy X-ray/γ-ray radiation. Although radiation hardness is a key property concerned in high-power physics,^59^ it shows the limited effect on common medical imaging.

#### (8) Proportionality

This parameter has an obvious impact on intensity identification ability, so linear scintillation response is required.

#### (9) Spatial resolution

It reveals the contrast ratio and the number of blurs (image sharpness) under a specific spatial frequency, which can be calculated by the modulation transfer function (MTF).^60^

### 3.2 Specific requirements for X-ray CT imaging

Integrated down-conversion scintillators are generally employed by modern digital X-ray imaging apparatuses and CT scanners to optically match the peak sensitivity window of the Si p-i-n photodiode matrix. The exposure dose has been the first concern, so the scintillators must have enough stopping power to absorb almost all of the incident X-ray energy. That is, for a given imaging resolution, the radiation dose is required to be as low as possible. For example, the thin ceramic scintillator screen (0.1–0.2 mm) can satisfy the lowest X-ray energy detection (around 20 keV in mammography). In dental radiography (around 60 keV) and full-body CT scanning (approximate 150 keV), a thicker scintillator screen is needed. With better transparency, mono-crystalline scintillation materials are thus employed instead of ceramic counterparts. Modern CT scanners need to record nearly 1000 slices per second, *via* memory effect, afterglow emission will cause unwanted ghost images, thus ultra-fast decay is required. Based on these special requirements in terms of clinic application, the key parameter criteria for scintillators used in X-ray CT imaging are summarized below:^13,61^

#### (1) High absorption coefficient

For a reliable reference for clinical diagnosis, the imaging contrast must reach a certain level, thus a certain amount of exposure dose cannot be avoided. Promotion in scintillating efficiency can remarkably cut down the necessary radiation dose for an acceptable CT image quality.^62^ To guarantee reasonable image quality at a suitable radiation dose exposure on the patient, the absorption coefficient should approach 100% at about 2 mm thickness.

#### (2) High light yield

For image noise elimination especially at the low signal level, light yield >20 000 photons per MeV is a particularly attractive scintillation parameter.^13^

#### (3) Fast decay

To track the signal variation produced under sub-0.5 s scan time, scintillating materials with ultra-fast decay time is required for X-ray CT detectors, generally <1 ms is acceptable.^61^ But in fact, to match the projection sampling rate (at ≥10 kHz) of modern CT scanners, the decay time should achieve at least 1–10 μs range.^13^

#### (4) Weak scintillation afterglow

This parameter is expressed by the percentage of X-rays that remain 3 ms after the turned-off of continuous X-ray radiation, with a desirable value of <0.1%.^13^ For CT applications, the elimination of afterglow is the most effective means to avoid sickle artifacts.

#### (5) Small thermal coefficient of light output

The thermal light coefficient reveals the variation in light yield under temperature fluctuation, ideally to be in the ±0.1% per °C range.^13^

#### (6) Matching radioluminescence spectrum

For CT imaging, Si-photodetectors are generally applied. In view of this, the ideal peak scintillation emission wavelength is within 400–900 nm to match the maximum spectral sensitivity of photodiode arrays.^63^

## 4. Scintillation materials

Due to the superior and tunable scintillating properties, rare earth (RE) doped nanocrystals are widely applied in X-ray imaging.^16,64^ For CT applications, by bandgap engineering or specific doping strategy, fast-decay scintillation in microsecond/nanosecond magnitude with weak afterglow can be achieved. The emission originates from the 5d–4f/4f–4f transition of doped RE ions are generally located in the visible light region (green-yellow), matching well with the sensitive readout range of common photodiodes. Due to the spin and parity allowed 5d–4f transition, intense scintillation emission can be produced from the Ce^3+^ center, with a relatively fast decay in nanosecond order. Eu^2+^, Er^3+^, Ho^3+^, Tm^3+^, and Pr^3+^ are also commonly used RE-dopants because of their wide emission bands (visible-NIR) that are generated by the fast 4f–4f transition in several microseconds.^52^

The energy resolution is identified as a crucial performance parameter required for practical X-ray scintillation imaging applications. Eu^2+^-doped halide scintillation materials such as SrI_2_:Eu^2+^ and CsBa_2_I_5_:Eu^2+^ not only exhibit high energy resolution but also have high LY because quenching will not happen though Eu^2+^ doping has achieved a high ionic concentration.^65–67^ Whereas the adverse reabsorption caused by self-absorption of surrounding Eu^2+^ will become worse if the doping concentration is too high. As a result, scintillation performance including LY, energy resolution as well as decay time will all be affected.^65^ A novel strategy has been reported to reduce the reabsorption effects by low-concentration co-doping ions such as Sm^2+^ that can absorb Eu^2+^emission but re-emit it in a higher NIR window.^68,69^ CsBa_2_I_5_:Eu^2+^, Sm^2+^ is one of the successful cases, by shifting the emission wavelength to NIR range, a relatively high energy resolution of 3.2% (under 662 keV γ-ray, APD detector) can be realized.^9^

Metal-halide perovskites are emerging photovoltaic materials, with tremendous light-conversion efficiency and adjustable luminous properties.^70–73^ Besides promising applications in solar cells,^74^ they are also potential scintillation materials for X-ray/γ-ray detection, among which lead halide perovskite is the most typical representative.^15,73^ However, the light output of this type of materials such as CsPbBr_3_ nanocrystal is only 21 000 photons per MeV,^73^ much lower than conventional scintillators like GOS:Pr (50 000 photons per MeV) and CsI:Tl (66 000 photons per MeV).^75^ Small stokes-shift and strong self-absorption are reported to be the main causes for the limited light yield of Pb-halide perovskite thin-films/nanocrystals.^76,77^ And from the perspectives of environmental protection and sustainable development, the practical applications of Pb-halide perovskites will be severely restricted due to the toxicity of Pb and the material instability.^78^ Thus, developing stable Pb-free perovskites with high PLQY becomes a new research hotspot.^27,77^ The summary of the key parameters for various scintillators from conventional materials to recent Pb-free perovskites in X-ray imaging has been indicated in Table 1.

**Table 1:** Summary of the key parameters for scintillators in X-ray imaging, from typical representatives to novel Pb-free perovskites

Scintillator	Density (g cm^−3^)	Peak emission (nm)	Light yield (ph per MeV)	Decay time (ns)	Afterglow	References
NaI:Tl^+^	3.67	415	38 000–55 000	250	—	11 and 12
BGO	7.13	480	8000–10 000	300	—	11 and 12
LYSO:Ce^3+^	7.1–7.2	420	30 000–33 000	45	—	11
CWO	7.9	495	28 000	5000	0.05% after 3 ms	13 and 14
GOS:Pr^3+^, Ce^3+^, F^−^	7.34	520	50 000	2400	<0.1% after 3 ms	13
GOS:Tb^3+^, Ce^3+^	7.34	550	50 000	6 × 10^5^	0.6% after 3 ms	13
CsPbBr_3_	—	525	21 000	8.09	—	73
Cs_3_Cu_2_I_5_:Tl^+^	—	440, 510	87 000	957, 717	0.17% at 10 ms	112
Cs_2_Ag_0.6_Na_0.4_In_1−*y*_Bi_*y*_Cl_6_	—	605–652	39 000 ± 7000	1	0.1% at 16 μs	27

### 4.1 Rare-earth doped scintillators

**Fig. 3:**
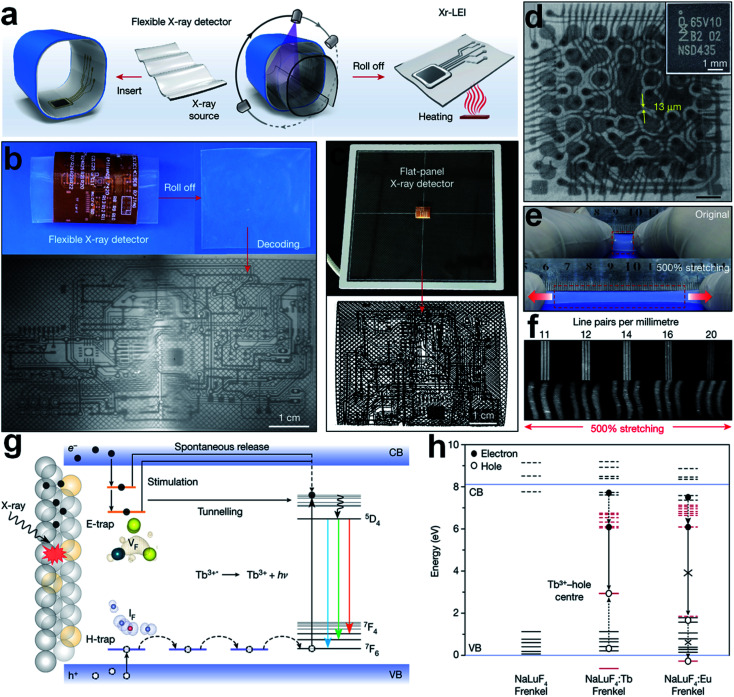
(a) Schematic description of three-dimensional (3D) electronic scintillation imaging through nanoparticles based stretchable X-ray detector. (b) X-ray luminescence extension imaging (Xr-LEI) of a 3D circuit board by the NaLuF_4_:Tb^3+^(15 mol%)@NaYF_4_ based flexible X-ray detector (50 kV, 80 °C). (c) X-ray imaging on the same electronic board by a traditional packaged flat panel detector. (d) Xr-LEI of inner circuits of an iPhone. Inset: digital photo of the same integrated circuits. (e) Photograph of the flexible scintillation layer. (f) High-quality Xr-LEI by applying the flexible X-ray detector. (g) Mechanism of long lifetime scintillation emitting of NaLuF_4_:Tb^3+^ nanocrystals. (h) Energy diagram of lanthanide 4f levels (in red) with reference to host bands (in black). Solid and dotted lines indicate occupied and empty states, respectively. Reproduced with permission.^64^ Copyright 2021 Springer Nature Limited.

Long-lasting phosphorescent materials can gradually de-trap the stored electrons for long afterglow emission, facilitating the development of flat-panel free detector for X-rays.^64^ Transition metal/RE doped inorganic oxides like W(vi)-doped ZnGa_2_O_4_:Cr^3+^ and Eu^3+^-doped NaGdF_4_ have already been applied for *in vivo* X-ray imaging, but they still exhibit relatively poor X-ray sensitivity.^79,80^ In addition, the manufacturing technology for crystal growth is complicated. In particular, for the formation of abundant electron trapping states within the host bandgap, high synthesis temperature over 600 °C is required.^81^ Benefit from the rapid development of nanotechnology, nanoscale lanthanide scintillators are successfully fabricated for X-ray conversion.^64,82^ Recently, a novel Tb^3+^-doped NaLuF_4_ nano-scintillator has been reported to have high X-ray absorption efficiency and super-long light emission lifetime, making it possible for flat-panel free X-ray 3D imaging for highly curved objects, see Fig. 3a–f.^64^ The outstanding X-ray conversion and long-term electron storage ability can be explained by the defects formation under high energy excitation (Fig. 3g and h). Small anions have migrated from their original lattice sites to interstitial sites, resulting in vacancy-interstitial defect pairs as Frenkel defects.^83^

**Fig. 4:**
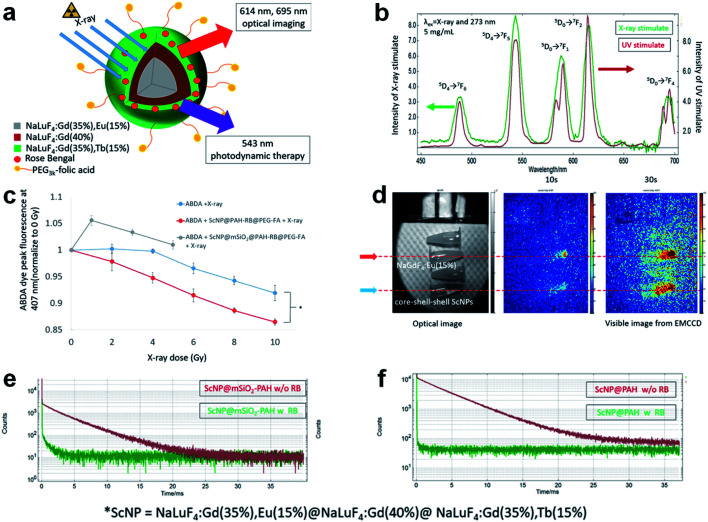
(a) Schematic description of novel RE-doped scintillation nanocomposite (core–shell–shell structured NaLuF_4_:Gd, Eu@NaLuF_4_:Gd@NaLuF_4_:Gd, Tb) for X-ray imaging and PDT. (b) Emission spectra of the core–shell–shell scintillator nanoparticle (ScNP) under X-ray/UV irradiation. (c) ^1^O_2_ generation test under X-ray radiation. (d) X-ray induced luminescence by a homemade fluorescence/computed tomography (FT-CT) device. (e) Exponential decay curves of Tb^3+ 5^D_4_ → ^7^F_5_ transition before and after the loading of Rose-Bengal: ScNP@mSiO_2_–poly(allylamine) and (f) ScNP–poly(allylamine). Reproduced with permission.^82^ Copyright 2018 American Chemical Society.

Another novel RE-doped scintillation nanocomposite (core–shell–shell structured NaLuF_4_:Gd, Eu@NaLuF_4_:Gd@NaLuF_4_:Gd, Tb) has been reported to have dual functions for PDT and X-ray imaging, as shown in Fig. 4a.^82^ Under X-ray irradiation, visible light emissions can be generated from the Tb^3+^ center (543 nm), stimulating the pre-loaded photosensitizer (Rose-Bengal). With the formation of ^1^O_2_, the target cancer cells will be killed efficiently (see Fig. 4b and c). Meanwhile, the incident X-ray can be down-converted to visible emission from the recombination center of Eu^3+^ (614 nm and 695 nm), making the scintillation imaging possible (see Fig. 4b and d). The decay time is sharply declined from 4243 μS to 29 μS with the loading of Rose-Bengal due to the energy transfer (ET) from Tb^3+^ to Rose-Bengal. But this rate of decay still cannot satisfy the requirement of high-speed CT scanning (see Fig. 4e and f).

### 4.2 All-inorganic Pb-halide perovskite scintillators

With an ultra-high solar-to-energy power conversion efficiency of over 20%, perovskites photovoltaic materials are recent research focus in solar cells,^84,85^ photoelectric sensors,^86^ X-ray/γ-ray detection.^87–90^ After a decade of development, depending on the innovation in film-growth technique and interface engineering, the photoelectric conversion efficiency of organic–inorganic perovskites-based solar cells has dramatically increased from 3.8% to 25.2%.^91–93^ Whereas, the intrinsic thermal instability of these hybrid perovskites, in organic–inorganic absorbing/organic charge-migrating layer, severely limits their extensive commercial applications.^94^ Fortunately, all-inorganic perovskites (such as CsPbI_3_ and CsPbI_2_Br) are identified that exhibited better thermal stability,^95^ attracting intense research attention in recent years. And the material stability under high-energy radiation is found to be promoted obviously when the organic cations (MA^+^ or FA^+^) in hybrid perovskites are substituted by Cs^+^.^88^ The high-*Z*_eff_ effect of Cs^+^ based perovskites exhibit better X-ray absorption capacity in equivalent thickness to organic cations based counterparts. In addition, the electrical resistivity of all-inorganic Pb-halide perovskites is generally higher than that of organic ones, which contributes to a lower dark current.^96^ Based on the factors mentioned above, all-inorganic perovskites are more promising materials for X-ray scintillation.

During the past three decades, the scintillating properties of all-inorganic Pb-halide perovskites have been deeply investigated.^26^ Early single-crystal CsPbCl_3_ exhibits a quite low light output of 320 photons per MeV, not capable of practical scintillation applications.^97^ Subsequently, single-crystal CsPbBr_3_ has been synthesized, with a high light-output (40 000–60 000 photons per MeV) under 12 keV X-ray excitation and an ultra-fast response (1 ns) at the low ambient temperature of 7 K.^98^ However, the evaluation reports on their room-temperature scintillation properties are very rare in early research. Since 2015, halide perovskite CsPbX_3_ (X= Cl, Br, I, or mixed-halide systems) nano-scintillators have been synthesized *via* hot-injection method, realizing a high PLQY up to 90%, a fast-decay of 1–29 ns, and tenable emission in the whole visible range (410–700 nm).^99^ From then on, all-inorganic Pb-halide perovskite nanocrystals have been intensely investigated for X-ray scintillation detection.

In terms of X-ray scintillating applications, material stability under high-power radiation is also a fundamental factor for medical detector construction. Various all-inorganic Pb-halide perovskites have attracted increasing research interests in scintillation applications due to their fast response and high detection efficiency to X-rays.^100^ Generally, bulk all-inorganic Pb-halide perovskites are manufactured by the Czochralski method, under an ambient temperature of over 1700 °C.^101^ Whereas, single-crystal perovskite scintillators are very sensitive to ambient humidity and exhibit limited stability in air.^67^ To achieve satisfactory X-ray scintillation performance, a low temperature below 130 K is needed.^1^ Benefiting from the development of nanotechnology, for further scintillation-property modification, researchers are inspired to shrink the material size down to the nanoscale.^54,102,103^ The emerging nanoscale perovskites are discovered that have good processability in the fabrication of scintillation thin-film, which is favorable for the construction of flexible devices.^103^ Besides, due to quantum and small-size effects, perovskite nano-scintillators exhibit distinctive electromagnetic and highly-expanded optical features.^104^ It should be also noted that on the one hand the material minification into nanoscale can bring about highly improved scintillation property but on the other hand it causes a decrease in effective mass density, and thus reduce the stopping power. Fortunately, researchers have found effective solutions for this problem by new material fabrication technology. For instance, under high-energy X-ray irradiation, the nano-scintillator CsPbCl_3−*X*_Br_*X*_ will easily decompose so it is not capable of long-term operation.^105^ Through blade coating technology, a large-scale all-inorganic perovskite film (CsPbBr_3_/Cs_4_PbBr_6_) has been successfully fabricated to function as an imaging screen, exhibiting good stability to resist radiation damage and humidity in air.^106^

**Fig. 5:**
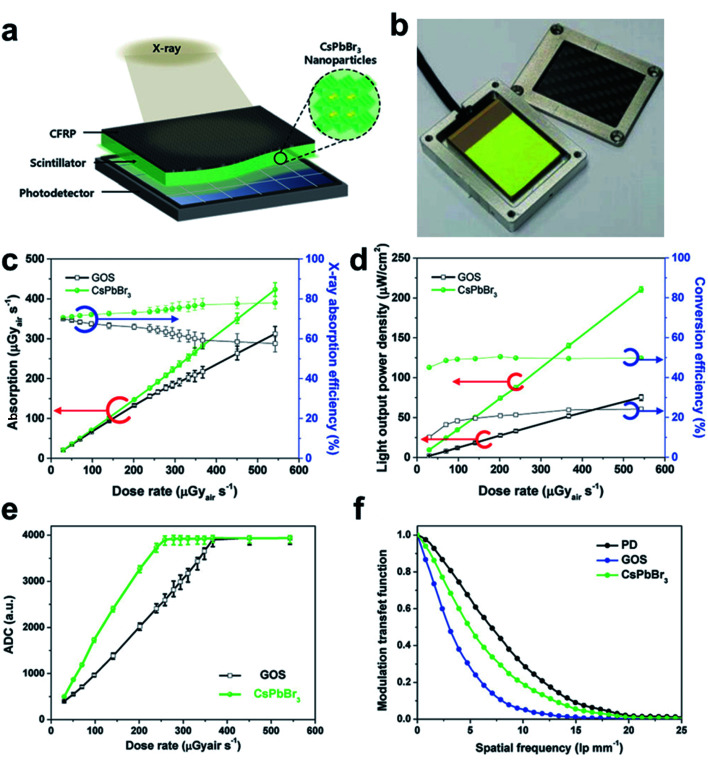
(a) Schematic CsPbBr_3_ perovskite nanoparticles-based X-ray detector. (b) Photograph of the CsPbBr_3_ perovskite nano-scintillator film on a Si-photodetector module. (c) Comparisons on X-ray absorption efficiency of CsPbBr_3_ perovskite nanocrystal and commercialized GOS scintillator. (d) Scintillation performance comparison for CsPbBr_3_ and GOS on light output power density and X-ray conversion efficiency. (e) Analog to digital converted (ADC) values under various X-ray dose rates by CsPbBr_3_ and GOS scintillation respectively. (f) Modulation transfer function of pristine Si-photodetector, CsPbBr_3_ perovskite nano-scintillator, and traditional GOS scintillator based X-ray detection. Reproduced with permission.^107^ Copyright 2018 Wiley-VCH.

Recently, a novel commercializable CsPbBr_3_ perovskite nano-scintillator with high-performance and low cost has been reported for non-destructive X-ray imaging (Fig. 5a and b).^107^ It has been identified that the proposed CsPbBr_3_ perovskite nanocrystal film has superior scintillation properties than conventional GOS film. As indicated in Fig. 5c and d, both the X-ray sensitivity and absorption efficiency of CsPbBr_3_ are much higher than that of GOS. We can also find that the light-yield power density and absorption efficiency for both samples are in a good linear correlation with the imposed dose rate. On the other side, it is obvious that the CsPbBr_3_ counterpart is more sensitive to X-ray radiation. The X-ray conversion efficiency of CsPbBr_3_ has reached 45–49%, more than twice of commercialized GOS. The practical scintillation performances of both films are also tested by applying them as scintillator layers in an X-ray detector. As seen from Fig. 5e and f, owing to its outstanding spatial resolution, higher image resolution can be obtained from CsPbBr_3_ nano-scintillator based X-ray detector than that sensed by GOS scintillation, particularly at relatively low radiation dose.

**Fig. 6:**
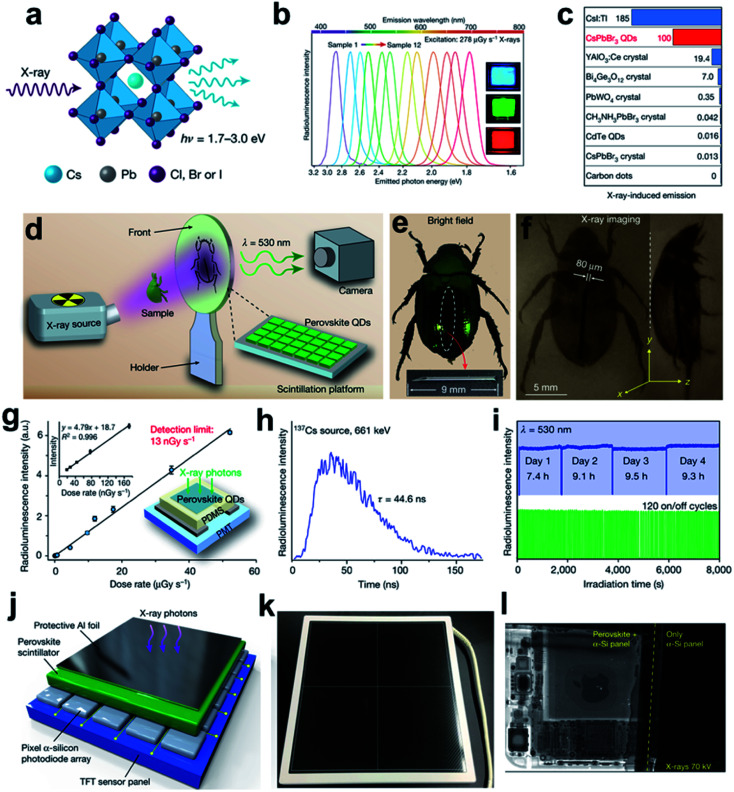
(a) Schematic description of X-ray radioluminescence (RL) produced in a cubic all-inorganic perovskite lattice. (b) Tunable emission wavelength of the CsPbX_3_ perovskite quantum dots under X-ray excitation (278 μGy S^−1^, 50 kV). (c) Comparisons of the optical sensitivity on different scintillators under 10 kV X-ray excitation. (d) Flow chart for real time X-ray scintillation bio-imaging. (e) Bright field and (f) X-ray imaging recorded by a common digital camera. (g) RL intensity of CsPbBr_3_ in response to various X-ray dose rates. Inset: RL intensity at low-dose rates (left) and schematic CsPbBr_3_ nano-scintillator thin-film based X-ray detector (right). (h) RL decay curves of CsPbBr_3_ nano-scintillator. (i) Photostability tests for CsPbBr_3_ nanocrystal under continuous and repeated cycles X-ray exposure. (j) Multilayered design of the flat panel X-ray imaging platform constituted by a thin-film transistor sensing panel, a pixelated α-Si photodiode array, a CsPbBr_3_ nano-scintillator thin film, and a protective Al-foil cover. (k) The packaged flat panel X-ray photodetector. (l) Comparison of X-ray images of an iPhone obtained with (left) and without (right) the perovskite CsPbBr_3_ nano-scintillator. Reproduced with permission.^15^ Copyright 2018 Springer Nature Limited.

Another successful perovskite CsPbBr_3_ nanocrystal scintillator has also been reported that have sharp and tunable light emissions over the visible spectrum range, making it capable of high-quality X-ray imaging in the common digital camera (Fig. 6a and b).^15^ Furthermore, under relatively low dose radiation of 5.0 μGy s^−1^, the optical sensitivity of CsPbBr_3_ nano-scintillator film (0.1 mm) still exhibits comparability to that of typical CsI:Tl bulk single crystal (5.0 mm). And the X-ray conversion efficiency is five or even dozens of times higher than those conventional scintillation crystals such as YAlO_3_:Ce, BGO, and PbWO_4_ (see Fig. 6c). In phase-contrast imaging application, since the stopping power of metal and insect-tissue for X-ray is very different, a high-contrast image of the beetle body with a metal needle inside can be obtained over the background of CsPbBr_3_ nano-scintillator substrate film (Fig. 6d–f). For clinical uses, exploring novel materials or methods for further decreasing the imposed dose rate with satisfactory imaging resolution is highly significant for the reduction of radiation injury.^37^ In this work, an ultra-low detection limit of 13 nGy s^−1^ has been achieved, with a radiation dose more than 400 times lower than that used in common X-ray diagnostics (Fig. 6g).^108^ Besides, with good photostability, the proposed nano-scintillator exhibits a relatively rapid response in 44.6 ns, indicating its potential in high-speed CT scanning (Fig. 6h and i). The suitability for the construction of a flat-panel X-ray detector has also been evaluated (Fig. 6j and k). The light scattering in nanocrystal thin-film substrate is much lower than that in typical micropillar/polycrystalline ceramic based bulk scintillators, which contributes to higher spatial resolution. As indicated in Fig. 6l, the inner structure of electronic circuit in an iPhone has been clearly imaged under low radiation dose (15 μGy). To sum up, this perovskite CsPbBr_3_ nanocrystal scintillator is a very promising candidate for future radiographic diagnosis under low-dose exposure.

### 4.3 Lead-free halide perovskite scintillators

As illustrated above, the novel Pb-halide perovskite nano-scintillators (CsPbX_3_) have exhibited their assets of facile manufacture, high conversion efficiency, high spatial resolution, good photo-stability and fast response.^15,89,107^ Whereas, these nanoscale scintillators still suffer relatively low light-output, poor stability, and the toxicity of Pb, which imposes a great restriction on their practical utilization in low-dose radiographic diagnosis.^27^ Recently, a series of Pb-free halide perovskite scintillators based on substitutional metal ions such as Cu,^31,76,109–112^ Bi,^32,113,114^ and double perovskites^27,29,115^ have revealed improved scintillation performance, featuring environmentally friendly fabrication. Among them, although Rb_2_CuBr_3_ (ref. 76) and Cs_2_NaTbCl_6_ (ref. 33) display high radio-luminescence output, the intense PL severely limits their applications in CT imaging. And the emission band of the proposed Rb_2_CuBr_3_ scintillator is located in the blue-light range, mismatching the peak sensitivity of a standard digital camera. Thus, the exploration of Pb-free halide perovskite scintillators characterized with high light output, short decay time, as well as applicable/tunable emission wavelength is deemed to be a promising research orientation.

**Fig. 7:**
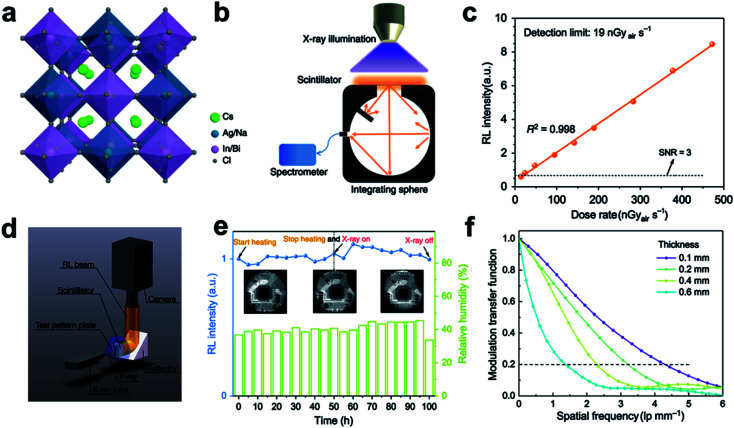
(a) Crystal structure of the novel Pb-free double perovskite (Cs_2_Ag_0.6_Na_0.4_In_0.85_Bi_0.15_Cl_6_). (b) Schematic radioluminescence (RL) spectra by an integrating sphere as well as a spectrometer with a constant distance from X-ray source to object. (c) RL intensity of the proposed double perovskite detected at low-dose rates. (d) Schematic X-ray imaging platform. (e) Integrated RL intensity of the proposed double perovskite under thermal treatment (50 h at 85 °C) followed by X-ray irradiation for another 50 h (12 μGy_air_ S^−1^, 50 kV). (f) Modulation transfer function curves of the proposed double perovskite wafers with different thicknesses. Reproduced with permission.^27^ Copyright 2020 Springer Nature Limited.

In a recent report, a novel Pb-free double-perovskite single-crystal Cs_2_Ag_0.6_Na_0.4_In_1−*Y*_Bi_*Y*_Cl_6_ (Fig. 7a) are synthesized and tested to have an improved light yield of 32 000–46 000 photons per MeV and a super short decay time of 1 ns when in Cs_2_Ag_0.6_Na_0.4_In_0.85_Bi_0.15_Cl_6_ form, due to the accelerating radiative recombination triggered by the appropriate concentration of Bi^3+^ dopant (*Y* = 0.15).^27^ This perovskite scintillator shows a good radiation stability as well as long-term thermal and chemical stability in air. High-resolution images of various objects can also be attained by low-dose radiation exposure (47.2 μGy S^−1^ for dynamic and 1 μGy for static imaging), indicating its potential applications in clinic radiography use (Fig. 7b–f).

**Fig. 8:**
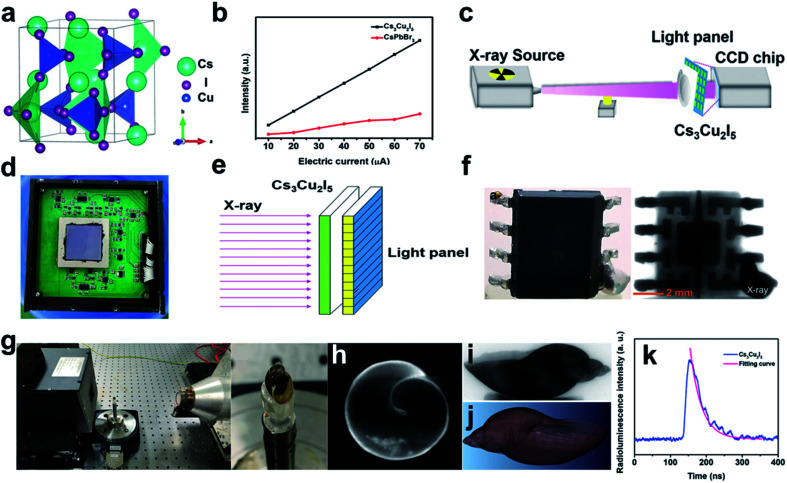
(a) Crystal structure of the novel Pb-free perovskite Cs_3_Cu_2_I_5_. (b) The comparison in LY of CsPbBr_3_ quantum dots and Cs_3_Cu_2_I_5_ nanocrystal under X-ray radiation. (c) Schematic description of the Cs_3_Cu_2_I_5_-based equipment for X-ray scintillation imaging. (d) The photograph of Charge Coupled Device (CCD) chip. (e) Schematic depiction of the Cs_3_Cu_2_I_5_-based X-ray scintillation imaging. (f) The image of chip obtained through the proposed device. (g) The proposed apparatus and the subject snail. The CT imaging apparatus includes an X-ray radiation source and the Cs_3_Cu_2_I_5_ scintillator-based detector. (h) The projection image of the subject snail. (i) The reconstructed slice image of the subject snail. (j) The volume rendering of the subject snail. (k) The RL decay curve of Cs_3_Cu_2_I_5_ nanocrystal. Reproduced with permission.^77^ Copyright 2021 Wiley-VCH.

Recently, low-dimensional especially zero-dimensional (0D) perovskite materials have addressed intense attention due to their outstanding scintillation properties for future optoelectronic and RL imaging applications. A 0D Pb-free Cs_3_Cu_2_I_5_ perovskite (see Fig. 8a) nanocrystal scintillator has been successfully synthesized in solution by a method at low-temperature, based on which a novel optical fiber panel has been manufactured for high-quality X-ray CT imaging.^77^ This Pb-free perovskite nano-scintillator has an adequate PLQY (59%) and extraordinary material stability in the ambient atmosphere for over three months. It also possesses a high scintillation LY, four times higher than the CsPbBr_3_ nanocrystal counterpart at 80 kV, 70 μA (Fig. 8b). To test its potential application in practical clinic imaging, a Cs_3_Cu_2_I_5_ nano-scintillator based X-ray detecting system has been constructed (Fig. 8c–e). A high-resolution projection image of the interior structure for a chip (Fig. 8f) and a three-dimensional CT image of a snail subject (Fig. 8g–j) can be obtained from this Cs_3_Cu_2_I_5_ scintillator-based X-ray detector, exhibiting its promising application prospect for commercial X-ray scintillation imaging. The Cs_3_Cu_2_I_5_ scintillator has a quite fast response, and a short decay time of 38.94 ns has been achieved (Fig. 8k), making it capable for clinic radiography including CT.

**Fig. 9:**
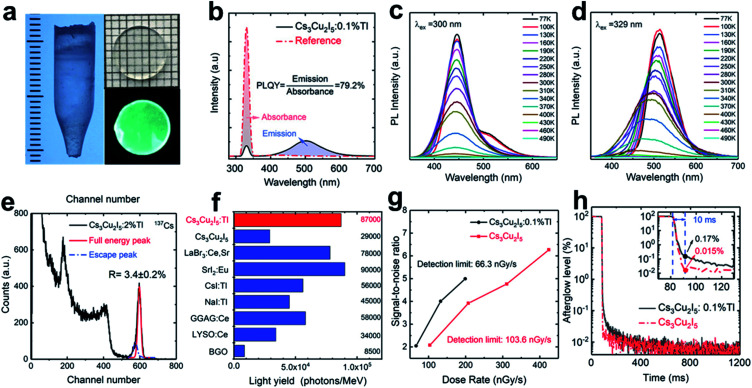
(a) A Cs_3_Cu_2_I_5_:Tl^+^ crystal ingot grown in 7 mm diameter as well as a 1 mm thick panel under natural (up) and UV light (down). (b) PLQY profile of the 0.1%Tl^+^ doped-Cs_3_Cu_2_I_5_ single crystal. (c and d) Temperature dependent photoluminescence spectra for 0.1%Tl^+^ doped-Cs_3_Cu_2_I_5_ (77–490 K): (c) STE and (d) Tl^+^-centre emission respectively. (e) Pulse height profile of 2% Tl^+^-doped Cs_3_Cu_2_I_5_ obtained from ^137^Cs radiation source. (f) Comparisons in absolute LY between Tl^+^-doped and pure Cs_3_Cu_2_I_5_, and other typical scintillation materials. (g) Comparison in SNR as a function of X-ray dose rate for 0.1%Tl^+^-doped and pure Cs_3_Cu_2_I_5_. (h) Afterglow curves for 0.1% Tl^+^-doped and pure Cs_3_Cu_2_I_5_.^112^ Copyright 2021 Wiley-VCH.

It has been also reported that the scintillation performance of low-dimensional Pb-free perovskite nanocrystal can be improved a lot by doping methods. In a recent report, Tl^+^ has been successfully doped into the host lattice of 0D perovskite Cs_3_Cu_2_I_5_ for the growth of crystal ingot (Fig. 9a), based on which a scintillator-based detecting platform are expected to be established for X-ray/γ-ray detection.^112^ Under X-ray radiation, the Cs_3_Cu_2_I_5_:Tl^+^ scintillator has an enhanced PLQY of 79.2% (Fig. 9b), and its RL includes a self-trapped exciton (STE) emission peaked at 440 nm (Fig. 9c) as well as a Tl^+^-centre emission peaked at 510 nm (Fig. 9d). With Tl^+^ doped, a steady-state scintillation LY of 150 000 photons per MeV can be obtained, approximately five times more than that of the undoped counterpart.^116^ An outstanding energy resolution of 3.4 ± 0.2% at 662 keV (Fig. 9e) and a high LY of 87 000 photons per MeV (Fig. 9f) have been also achieved. When the signal-to-noise ratio (SNR) is set to be two, the X-ray detection limit can be reduced from 103.6 nGy_air_ S^−1^ (undoped) to 66.3 nGy_air_ S^−1^ (Tl^+^ doped) (Fig. 9g), much lower than the requirement of 5.5 μGy_air_ S^−1^ in clinic diagnosis by X-ray imaging.^108^ However, as indicated in Fig. 9h, the afterglow emission (0.17% at 10 ms) of Tl^+^-doped Cs_3_Cu_2_I_5_ single crystal is not preferable for high-speed CT scanning imaging.

## 5. Challenges and outlook

In this work, we have comprehensively summarized the recent research achievements in all-inorganic perovskite nanocrystal for X-ray scintillation imaging. The significant advances cover the traditional heavy-metal based compounds (NaI:Tl^+^/CsI:Tl^+^, CWO, BGO, LSO, and LYSO), the latest perovskite scintillators CsPbX_3_ (X= Cl, Br, I, or mixed-halides) as well as the Pb-free perovskite scintillators, which especially emphasizes the clinical applications. In terms of material morphology, nanoscale perovskites exhibit superior scintillation performance at room temperature when compared with bulk single crystals. The rapid development of nanotechnology further boosts the modification in X-ray scintillation behaviour for perovskite nano-scintillators, and then realizes satisfactory energy accumulation under low-dose exposure. The scintillation properties of perovskite nano-scintillators can be tuned by doping, bandgap engineering, or other material modification techniques to satisfy various application scenarios, but challenges still remain especially in the aspect of the stability in air and the toxicity of Pb^2+^.

Encapsulation operations are essential to consider for maintaining the long-term stability of halide-perovskite nanomaterials in ambient air. To address the pollution problems caused by poisonous Pb^2+^, a series of Pb-free halide perovskites like A_3_Bi_2_X_9_ (A = Cs^+^ or Rb^+^), Rb_2_CuBr_3_, Cs_2_AgBiBr_6_, and Cs_2_NaTbCl_6_ have been successfully synthesized to serve as potential substitutes for X-ray scintillation. But unfortunately, the scintillation performances of these Pb-free perovskites are still not competitive to that of Pb-halide perovskites. Consequently, persistent attempts are made to promote their scintillation performance, including trials on new crystal-growth techniques and developments on novel perovskite nanocrystal with new substitutes for Pb^2+^. At present, the halide-perovskite nanocrystal-based scintillation devices are still staying in laboratory-scale manufacturing. With a refractive index of approaching 1.0 for the general lens, X-rays are very difficult to be focused. Thus, manufacturing a large-area scintillation wafer/thin-film layer comparable to the size of the imaged subject is essential.^117^ In addition, the material reproducibility and toxicity removal technology of halide-perovskite scintillators also become recent research hotspots that ultimately orientate to realize commercialized production.

In typical X-ray detectors, the scintillation layer is required to be thick enough for sufficient absorption on incident radiations. As for X-ray imaging on nonplanar surfaces, the flexibility under a certain thickness is another key criterion for building the scintillation layer of detection devices. The fabrication of a large-sized scintillation layer with good flexibility can be solved by the successful preparation of perovskite films, whereas, maintaining stable material quality under adequate thickness is still a tough technical problem. For instance, for adequate X-ray absorption (22 keV) by (NH_4_)_3_Bi_2_I_9_ layer, a thickness of at least 2 mm is required, but current film-deposition technology cannot satisfy this material requirement.^118^ Thus, maintaining material thickness and flexibility in the large-area film is a breakthrough point for the further advancement of perovskite scintillators.

Due to the high penetrating power of NIR light in animal organs/tissues, shifting the emitting light of contrast medium to the higher biological window has been a research hotspot for high-penetration bio-imaging and photodynamic therapy (PDT).^37^ Down-converting incident X-ray to NIR emission will also be a promising research area to be explored in practical scintillation applications especially in high-radiation fields. The Cherenkov light appearing in UV/visible region will become a severe noise signal if the X-ray scintillating emission locates in the same spectral range, and what's worse, the original colour of optical fibres will gradually be changed due to the long-term absorption of UV/visible light.^119^ Thus, finding ways to develop novel NIR emission scintillators to avoid radiation damage and reduce the noise signal is of great significance for commercialized applications. Besides co-doping methods, the native point defects tuned NIR emission is also very promising for scintillation wavelength control if the electron-transition energy gaps in the NIR range between defect states can be precisely predicted by computational simulation on electronic properties.^37^

The self-absorption effect is another adverse factor that causes severe reduction of light output. An enlarged Stokes shift will lead to a better separation between the absorption and emission spectrum, offering us an effective method to restrain the self-absorption. For instance, the CsPbBr_3_ nanocrystal can serve as a sensitizer for organic dyes with large Stokes shift to realize a scintillation performance that almost eliminates the self-absorption effect, but unfortunately suffers a low light output of 9000 photons per MeV.^120^ Thus, finding ways to improve the light output of the perovskite nanocrystal sensitized organic dyes will be another research hotpot. Doping technology has been widely applied for the modulation of perovskite scintillators, by which the Stokes shift can also be enlarged.^121^ Relying on the ET process, the energy states of transition-metal/RE doping ions can act as the recombination center to produce scintillation emission with the wavelength corresponding to the transition gap.^37^ Addition to these doping levels, the native defect states within the bandgap also exhibit the potential to control the scintillation performance.^37,64,122^ The electronic properties of these intrinsic defects calculated through density functional theory can serve as a reliable theoretical instruction for future studies on defect-tuned scintillators.

## Conflicts of interest

There are no conflicts to declare.

## Supplementary Material
